# Advances in Ferroptosis Research: A Comprehensive Review of Mechanism Exploration, Drug Development, and Disease Treatment

**DOI:** 10.3390/ph18030334

**Published:** 2025-02-26

**Authors:** Haojie Wang, Yuanyuan Xie

**Affiliations:** 1 College of Pharmaceutical Science, Zhejiang University of Technology, Hangzhou 310014, China; 2 Collaborative Innovation Center of Yangtze River Delta Region Green Pharmaceutical, Zhejiang University of Technology, Hangzhou 310014, China; 3 Key Laboratory for Green Pharmaceutical Technologies and Related Equipment of Ministry of Education, Hangzhou 310014, China

**Keywords:** ferroptosis, mechanism, inducer, inhibitor, disease treatment

## Abstract

In recent years, ferroptosis, as an emerging modality of programmed cell death, has captured significant attention within the scientific community. This comprehensive review meticulously canvasses the pertinent literature of the past few years, spanning multiple facets. It delves into the intricate mechanisms underpinning ferroptosis, tracks the evolution of its inducers and inhibitors, and dissects its roles in a diverse array of diseases, as well as the resultant therapeutic implications. A profound exploration is conducted of the functional mechanisms of ferroptosis-related molecules, intracellular pathways, metabolic cascades, and signaling transduction routes. Novel ferroptosis inducers and inhibitors are introduced in detail, covering their design blueprints, synthetic methodologies, and bioactivity profiles. Moreover, an exhaustive account is provided regarding the involvement of ferroptosis in malignancies, neurodegenerative disorders, cardiovascular ailments, and other pathologies. By highlighting the pivotal status and potential therapeutic regimens of ferroptosis in various diseases, this review aspires to furnish a thorough and profound reference framework for future investigations and clinical translations in the ferroptosis domain.

## 1. Introduction

In recent years, ferroptosis, which represents a form of programmed cell death reliant on iron, has been drawing increasing attention. Characterized by the build-up of iron and lipid peroxidation, it plays a significant part in a wide range of physiological and pathological processes that simply cannot be overlooked. As research progresses further, ferroptosis has emerged as a prominent focus in life sciences, paving the way for novel concepts and approaches to the treatment of assorted ailments. In the field of ferroptosis research, there are many relevant studies, and research directions are complex and scattered. For example, some studies focus on key regulatory targets, inducers, and inhibitors in the metabolic pathway of ferroptosis [1], while others focus on hot topics related to ferroptosis in synthetic lethal research [2] and the effects of specific compounds on iron overload diseases [3].

Ferroptosis is closely related to a variety of diseases, such as diabetes [4], neurodegenerative diseases [5,6,7], and cancer [8]. In-depth analysis of its mechanism of action in these diseases is of great significance for the development of targeted and effective treatment strategies. Taking diabetes as an example, clarifying the specific mechanism of ferroptosis in the process of pancreatic beta cell death can provide a solid theoretical basis for the development of therapies that protect beta cell function and delay the progression of diabetes and its complications. In cancer research, understanding the relationship between ferroptosis and immunotherapy can provide a new direction for the optimization of cancer immunotherapy regimens.

At this stage, a series of ferroptosis inducers and inhibitors have been discovered, but there is still much room for improvement in terms of drug efficacy and safety. The review can systematically summarize the existing drug research and development results, such as screening ferroptosis inhibitors from FDA-approved drugs [9], summarizing the mechanism of action of natural and synthetic small-molecule ferroptosis inhibitors in detail. These summaries help researchers clarify the status and shortcomings of drug development, provide innovative ideas and directions for subsequent drug development work, and effectively promote the drug development process in a more efficient and safer direction.

**Figure 1:**
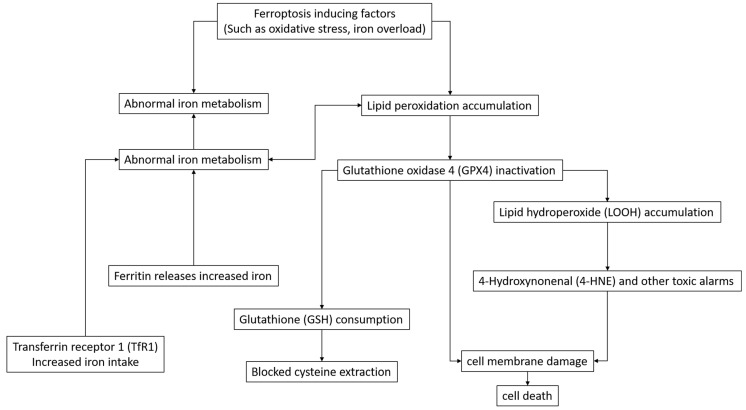
Schematic of the signaling pathways, molecules, and processes in the ferroptosis process.

This review provides an in-depth and comprehensive account of the mechanism of ferroptosis, including its intricate relationship with iron metabolism, lipid metabolism, and amino acid metabolism, as well as the regulatory mechanisms of related genes such as *Nrf2*, *FSP1*, *VDACs*, and *p53* [7]. In addition to *Nrf2*, *FSP1*, *VDACs*, and p53, there are other genes associated with ferroptosis. For example, the glutathione peroxidase 4 (*GPX4*) gene, whose expression product plays a key role in inhibiting lipid peroxidation and regulating ferroptosis; the cystine transporter SLC7A11-related gene, which affects cystine uptake and then participates in the regulation of ferroptosis; the transferrin receptor 1 (*TfR1*) gene, whose expression changes are closely related to iron uptake and ferroptosis process. At the same time, the differences and connections between ferroptosis and other cell death patterns are carefully analyzed, as discussed in [6], so as to gain a deeper understanding of its unique position in cellular physiology and pathological processes and provide a theoretical basis for precise interventions for ferroptosis. The existing ferroptosis inducers and inhibitors are systematically summarized, covering their classification, mechanism of action, and specific applications in disease treatment. For example, one study lists commonly used and newly developed inducers and inhibitors in detail [1], further analyzing their mechanisms of action and efficacy in diseases [6]. In addition, many challenges faced in the drug development process, such as drug selectivity, toxicity, and in vivo stability, have also been discussed in depth, and corresponding solutions have been proposed based on these issues, providing strong guidance for the optimal design of subsequent drugs. Several studies provide a comprehensive review of the application status of ferroptosis in the treatment of various diseases, such as a combined application strategy and effect evaluation of immunotherapy in cancer treatment [8], the protective mechanism and potential therapeutic value of pancreatic beta cells in diabetes treatment [4], the potential and application prospects of neuroprotection in neurodegenerative diseases [5,6,7], and an exploration of potential therapeutic effects in other diseases such as post-COVID-19 syndrome [10]. An in-depth analysis of the specific strategies, therapeutic effects, and existing problems of using ferroptosis in the treatment of different diseases provides a practical reference for clinical treatment. At the same time, we should pay close attention to new trends in the field of disease treatment, such as the combination therapy strategy of targeting ferroptosis [2,11], etc., to provide forward-looking ideas and directions for future research work. Ferroptosis is a regulated form of cell death, and its process involves multiple signaling pathways, molecules, and complex processes. Inducing factors such as oxidative stress and iron overload can trigger iron metabolism disorders and lipid peroxidation accumulation. Abnormal iron metabolism promotes an increase in iron ions. On the one hand, the uptake of iron through transferrin receptor 1 increases, and on the other hand, the release of iron from ferritin increases. The increased iron ions further exacerbate lipid peroxidation, leading to the inactivation of glutathione peroxidase 4, which results in the significant consumption of glutathione and blocks the uptake of cysteine. After glutathione peroxidase 4 is inactivated, lipid hydroperoxides cannot be metabolized normally and accumulate, and toxic aldehydes such as 4-hydroxy-2-nonenal are generated. Eventually, the cell membrane is damaged, triggering cell death (Figure 1).

## 2. Study on the Mechanism of Ferroptosis

### 2.1. Molecules and Pathways Related to Ferroptosis

Ferroptosis plays a key role in the initiation, execution, and regulation processes. At the initiation stage, changes in the intracellular environment trigger ferroptosis. During execution, reactive oxygen species destroy cellular components and promote cell death. In terms of regulation, many genes and signaling pathways work together to control the process of ferroptosis and maintain cellular homeostasis [1,4,7,12,13,14,15,16,17,18,19,20,21,22,23,24,25,26,27,28,29,30,31]. Menin-MLL inhibitors, such as MI-463, can induce ferroptosis in cancer cell lines. When used in combination with genofin, it can synergistically enhance the death of cancer cells, indicating its significance in the initiation process of ferroptosis. Menin-MLL inhibitors such as MI-463 can induce ferroptosis in cancer cell lines and in combination with genofin can synergistically enhance cancer cell death, suggesting that menin-MLL is important in the initiation of ferroptosis [17]. The p53 apoptosis-stimulating protein inhibitor (iASPP) inhibits ferroptosis and alleviates acute lung injury induced by intestinal ischemia/reperfusion. The protection mediated by it relies on the Nrf2 signaling pathway. This indicates that this protein plays a vital role in the regulation of ferroptosis. Specifically, when cells are confronted with stimuli that may trigger ferroptosis, relevant mechanisms activate the Nrf2 signaling pathway. This activation upregulates the expression of a series of antioxidant genes, thereby enhancing the cells’ resistance to oxidative stress and inhibiting the occurrence of ferroptosis. This fully demonstrates the crucial position of this protein in maintaining cellular homeostasis and regulating the ferroptosis process [20]. The NUPR1 inhibitor ZZW-115 can induce mitochondrial morphological changes, leading to ROS production and cell death, its mediated mitochondrial cell death can be rescued by Fer-1, and TFAM can be regarded as an antagonist of ferroptosis [15]. The loss of activity of glutathione peroxidase 4 (GPX4) leads to the accumulation of lipid hydroperoxides, which triggers ferroptosis. Powerful GPX4 inhibitors such as compound **26a** can induce ferroptosis, which has potential application value in cancer treatment [27]. The cystine transporter SLC7A11 of System Xc^−^ is part of the regulation of ferroptosis in some cancer cells [1]. In addition, in other disease-related studies, such as on ischemic heart disease, ferroptosis-specific inhibitor ferrostatin-1 alleviates cardiomyocyte redox imbalance through the Nrf2/ARE pathway, inhibiting ferroptosis [32]. In Parkinson’s disease, the polyphenol compound thoninigianin A in natural plant foods inhibits ferroptosis in dopaminergic cells by activating the Nrf2-related cellular protective system [23]. These studies demonstrate the complex regulatory role of different molecules in the process of ferroptosis, providing an important theoretical basis. One of the protective pathways for ferroptosis is the Nrf2 signaling pathway, but the protective pathway for ferroptosis is not unique. The Nrf2 signaling pathway can inhibit ferroptosis by regulating the expression of a series of antioxidant genes, thus enhancing the antioxidant capacity of cells and inhibiting lipid peroxidation. In addition, there are other pathways that also play a protective role in ferroptosis, such as the glutathione peroxidase 4 (GPX4) related pathway.

Dysfunction of the mitochondria is closely related to ferroptosis, and a variety of related processes are involved [33,34,35,36]. In a study of acute cerebral hemorrhage (ICH), it was found that mitochondrial damage was significant, and it showed a morphology like ferroptosis under an electron microscope. Ferroptosis has distinct morphological features, such as mitochondria shrink, increased membrane density, and loss of cristae, which may even involve outer-membrane rupture. The plasma membrane remains relatively normal until late-stage rupture, and other organelles like the endoplasmic reticulum and Golgi may swell, while the nucleus stays intact. Mitochondrial damage induced by rotiflin (Rot), a special inhibitor of mitochondria, produces CARM (coactivator-associated arginine methyltransferase) dose-dependent toxicity in primary nerve cells, inhibiting cell nerve activity. This suggests that ICH-induced mitochondrial dysfunction is closely related to ferroptosis in nerve cells. Studies on the specific mechanism of mitochondria in ferroptosis have shown that the fat-soluble organic peroxide tert-butyl hydroperoxide (TBH) induces iron apoptotic cell death; its combination with iron reduces mitochondrial swelling, inhibits oxidative phosphorylation, and stimulates NADH oxidation, while phospholipase activation, lipid peroxidation, and mitochondrial permeability transition pore (MPTP) openings are involved in mitochondria-dependent ferroptosis, and they play a role in different stages of membrane damage [34]. In tumor research, MP-HJ-1b, as a novel microtubule inhibitor, like colchicine, can promote ferroptosis in HeLa cells, such as by increasing reactive oxygen species and malondialdehyde levels and reducing glutathione levels. MP-HJ-1b also downregulates SLC7A11 and GPX4 to enhance the classic ferroptosis pathway [35]. In addition, nutmeg inhibits glioblastoma (GBM) growth in a Slug-dependent manner through EMT-mediated ferroptosis, which inhibits NF-κB signaling activation by blocking p65 protein phosphorylation and induces ferroptosis through the Slug-SLC7A11 signaling pathway [37]. These studies further confirmed the close connection between mitochondrial-dysfunction-related processes and ferroptosis from different disease perspectives, providing an important basis for further exploring the treatment of related diseases.

Ferroptosis has attracted much attention since it was first described in 2012 [38]. It has both links to and differences from apoptosis and necrosis, and its unique characteristics and regulatory mechanisms are gradually being revealed. In terms of its relationship with apoptosis, studies have shown that although mitochondrial oxidative stress is involved in both ferroptosis (e.g., erastin induction) and apoptosis (e.g., rotenone induction), the two are different cell death pathways [39]. The ferroptosis inhibitor oxindole–curcumin hybrid compound **GIF-2165X-G1** is selective for rotenone-induced apoptosis but does not affect the apoptosis process. In association with necrosis, nigretin has been characterized as a dual inhibitor of necrosis and ferroptosis cell death pathways, and it inhibits necrosis induced by tumor necrosis factor-alpha and ferroptosis induced by various small molecules [40]. **KW-2449**, a necrosis inhibitor, also prevents ferroptosis by targeting the autophagy pathway, and the classical necrosis inhibitor Necrostatin-1 also inhibits ferroptosis [41]. In addition, in neurological disease-related studies, the cell death pathway is regulated by oxidation/ferroptosis. **Cannabinol** (**CBN**) and its analogs have been found to be effective inhibitors of oxidation/ferroptosis. Related studies have identified its key molecular scaffolds and emphasized the combination of in vitro cell detection and in vivo studies of Drosophila models [42]. These research findings help us gain a deeper insight into the intricate connection between ferroptosis and other cell death modalities, laying a crucial foundation for therapy for relevant diseases.

Ferroptosis belongs to a regulatory cell death type catalyzed by iron. It is initiated when the build-up of lipid peroxidation products and reactive oxygen species reaches levels that are lethal, setting off this cell death process. It is of remarkable significance in cancer treatment and other aspects, and transcription regulators play a crucial role in fine regulation [13,43]. In hepatocellular carcinoma (HCC) studies, **CARM1** has been found to be a key inhibitor of ferroptosis [13]. When **sorafenib** induces ferroptosis as a standard drug for HCC, CARM1 depletion significantly exacerbates this process. The underlying mechanism behind this phenomenon involves CARM1-catalyzed **H3R26me2a** on the glutathione peroxidase 4 promoter, which leads to the transcriptional activation of this gene, thereby inhibiting ferroptosis. Sorafenib treatment can also induce the transcription of CARM1 through the MDM2-p53 axis. On the other hand, BRD4, as an epigenetic reading and cancer therapeutic target, has a controversial role in ferroptosis, but studies have shown that BRD4 inhibition has a significant effect on ferroptosis [43]. For cells such as HEK293T and HeLa, BRD4 inhibition greatly enhances erastin-induced ferroptosis, and BRD4 knockout also promotes cell death. BRD4 inhibitors such as **JQ-1** and **I-BET-762** or **BRD4** knockout can cause a large buildup of reactive oxygen species (ROS) within cells. At the level of gene expression, BRD4 inhibition has distinct impacts on genes related to ferroptosis within diverse cell types. The regulation of ferroptosis by these transcriptional regulators provides important research directions and potential strategies for the treatment of related diseases.

### 2.2. Studies on Metabolic Pathways Related to Ferroptosis

**Figure 2:**
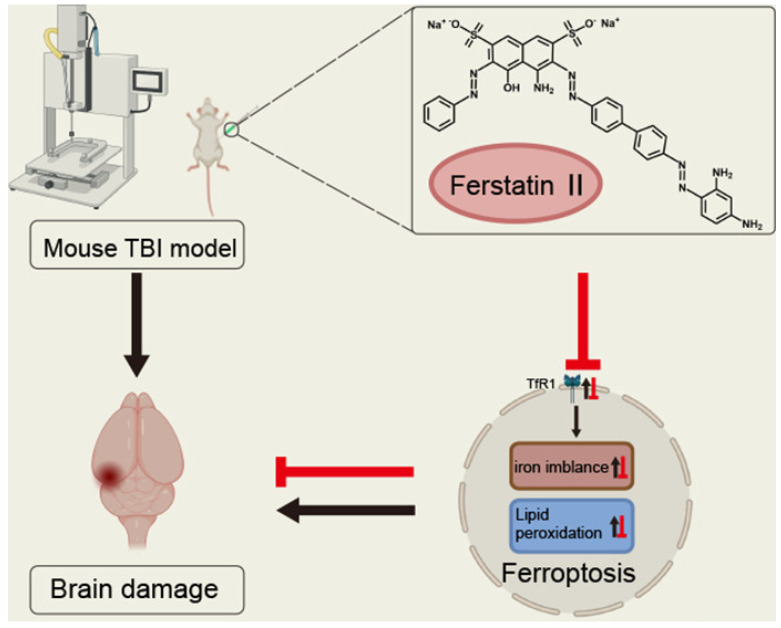
Schematic diagram of an iron uptake inhibitor, exerting neuroprotection against traumatic brain injury by suppressing ferroptosis—reported by Cheng et al. [47]. Reproduced with permission from Ref. [47]. Copyright 2022, American Chemical Society.

The metabolism of iron, lipids, amino acids, and glutathione exerts crucial regulatory functions during the development of ferroptosis. Any disruptions within these metabolic pathways are intricately associated with the onset and advancement of ferroptosis [3,4,6,7,18,21,23,28,44,45]. In terms of iron metabolism, iron overload can lead to cell damage and death through various pathways. For example, iron overload is one of the main causes of cardiomyopathy, and ferroptosis inhibitors may become a potential treatment [21,46]. At the same time, changes in iron-metabolism-related proteins also affect the process of ferroptosis, such as the increased expression of *transferrin receptor 1 (TfR1)*, which is associated with ferroptosis after traumatic brain injury [47] (Figure 2). Lipid metabolism is crucial in ferroptosis. Some studies have found that natural antioxidants such as dithiothionone in cruciferous vegetables can inhibit ferroptosis by modulating lipid-peroxidation-related processes; reduce intracellular reactive oxygen species and lipid peroxidation levels; and upregulate the manifestation of the iron storage protein ferritin, thus reducing the total unstable iron pool [3]. In Ewing sarcoma cells, carbonic anhydrase II (CAII) inhibitors can trigger ferroptosis by downregulating FTH1 while affecting the cell’s amino acid metabolism, thereby regulating the occurrence of ferroptosis [48]. Glutathione metabolism plays a central role in the regulation of ferroptosis. Moreover, glutathione peroxidase 4 (GPX4) is a key enzyme that regulates ferroptosis. Its activity depends on glutathione, which converts iron-dependent lipid hydroperoxides into lipid alcohols and inhibits lipid peroxidation. When GPX4 activity is inhibited, as in some cancer cells, it triggers ferroptosis [1,4,6,7,12,13,16,18,19,21,23,28,31,45]. In Parkinson’s disease, natural compounds such as **thoninigianin A** increase heme oxygenase-1 (HO-1) protein levels by activating a related pathway and modulating glutathione metabolism, thereby inhibiting ferroptosis [23]. In leukemia research, ferroptosis inducers such as **erastin** and **RSL3** inhibit leukemia cell proliferation by affecting glutathione metabolism, while ferroptosis inhibitors can rescue this inhibitory effect [18]. These metabolic pathways are interrelated and affect each other, providing potential targets and intervention directions. Energy metabolism disorders and increased oxidative stress caused by mitochondrial dysfunction are also related to ferroptosis.

### 2.3. Ferroptosis-Related Signaling Pathways

NF-κB, Nrf2, MAPK, PI3K/AKT, and other signaling pathways are of great significance in ferroptosis, profoundly affecting its occurrence and development [4,7,13,14,20,23,25,28,43,49,50,51,52]. In the NF-κB pathway, hepatic nuclear factor kappa B-induced kinase can promote oxidative stress and ferroptosis in hepatocytes, reflecting its regulation of the cellular redox state [52]. The Nrf2 pathway plays a role in multiple processes, such as in iron overload cardiomyopathy; related drugs can affect cellular redox balance and ferroptosis by regulating Nrf2 [21]. In the MAPK pathway, an inhibitor of SB202190 can inhibit ferroptosis in retinal ganglion cells and regulate cell survival [53]. The PI3K/AKT pathway regulates ferroptosis-related protein synthesis, and MEK inhibitor-resistant cells inhibit ferroptosis through this pathway in ovarian cancer [51]. These signaling pathways are intertwined to jointly regulate cellular redox, inflammation, and survival and have a profound impact on ferroptosis.

In hypoxic environments, the HIF-α signaling pathway is crucial for the regulation of ferroptosis [22,37,54]. For example, in glioblastoma, roxastat amplifies HIF signaling to induce ferroptosis and inhibit tumor growth. The mechanism is related to HIF-2α upregulating lipid regulatory gene activation and enhancing lipid peroxidation [22]. Nutmeg induces ferroptosis in GBM cells through the Slug-SLC7A11 signaling pathway by blocking NF-κB signaling activation, and the role of the HIF-α signaling pathway in it deserves further study [37]. Studies suggest that the HIF-α signaling pathway significantly affects the sensitivity of cells to ferroptosis.

In cancer treatment, the interaction of WEE1 inhibitors with ferroptosis-related signaling pathways has shown important significance [49,51]. P53 deficiency affects the sensitivity of NSCLC to WEE1 inhibitors, and WEE1 inhibitors induce intracellular lipid peroxidation. At the same time, in ovarian cancer, the dual inhibition of MEK inhibitors and the mTOR/4EBP1 signaling pathway can play a therapeutic role by modulating ferroptosis, providing a new idea for combination therapy. When breast cancer cells become resistant to FOXM1 inhibitors, ferroptosis-related signaling pathways are changed [19]. Pathways that inhibit ferroptosis in drug-resistant cells are activated, enhancing cell survival and drug resistance. The synergistic effect of low-dose FOXM1 inhibitors and ferroptosis inducers can reduce cell viability, providing a new direction for overcoming FOXM1 inhibitor resistance.

## 3. Ferroptosis Inducers: New Synthesis and Old Compound Activity

### 3.1. Design and Synthesis of Novel Ferroptosis Inducer

To effectively induce ferroptosis, many studies have been carried out based on multiple key targets [12,27,54,55,56,57,58,59,60,61,62,63,64,65]. For example, in RSL3 target-related studies, powerful inducers such as compound **26a** have been developed, which can effectively inhibit GPX4 activity and induce ferroptosis, and have shown the potential to inhibit tumor growth in mouse models [27]. For the ML162 target, it was found that it does not directly inhibit GPX4 but instead inhibits TXNRD1, which prompts a reassessment of its mechanism of action in ferroptosis [55]. In addition, a variety of novel compounds have been designed and synthesized around the GPX4 target, such as **ferrocene** plus GPX4 inhibitors, which induce ferroptosis by modulating GPX4 activity, providing new strategies for cancer treatment [12,57,58,59,60,64,65]. These research results provide more effective tools for the precise regulation of ferroptosis.

In the field of ferroptosis research, the exploration of novel compounds such as **ferrocene** plus GPX4 inhibitors is of great significance [59]. These compounds have verified the inhibitory effect of GPX4 and the ability to induce ferroptosis at the cellular level with a rationally designed strategy, which not only promotes the development of anti-cancer therapies but also opens new ideas and methods for the development of new ferroptosis inducers. 1,8-Naphthalimide piperazinamidobenzenesulfonamide derivatives have attracted much attention as novel ferroptosis inducers [60]. Their representative compound, **9o**, has great potential in the treatment of triple-negative breast cancer. It has strong inhibitory activity against carbonic anhydrase IX and is highly selective. It can induce tumor cell apoptosis and ferroptosis, effectively inhibit tumor growth and metastasis, and bring new hope for specific cancer treatments. Piperine derivatives have made new progress in anti-cancer research [30]. Among them, compound **H19** has exhibited strong inhibitory activity, which could play an anti-cancer effect by inducing ferroptosis, and its cytotoxicity could be reversed by ferroptosis inhibitors. This finding indicates that piperine derivatives have ferroptosis-inducing activity, providing a valuable reference for the development of inducers from natural products.

### 3.2. Ferroptosis-Induced Activity of Known Compounds

**Gliflurazine**, **dihydroartemisinin**, **JKE1674**, **RSL3**, **ML162**, and other compounds have achieved many results in the induction of ferroptosis and its mechanism, laying a theoretical foundation for related applications [8,19,43,49,51,58,62,66,67,68,69,70]. **Gliflurazine** can restore GPX4 expression in skeletal muscle cells in a high-sugar environment, inhibit ferroptosis, and enhance cell survival and paracrine function, and it has potential value in the treatment of diabetic complications [71]. **Dihydroartemisinin** and **JKE1674** work synergistically with FOXM1 inhibitors to reduce breast cancer cell viability, induce ferroptosis, and overcome FOXM1 inhibitor resistance [19]. **RSL3** induces ferroptosis in a variety of cancer cells, but its sensitivity varies in different cell lines. For example, in intracavitary breast cancer, it is associated with the HER2 pathway [68]. **ML162** has been found to be a TXNRD1 inhibitor, and the mechanism of its induction of ferroptosis needs to be re-evaluated [55]. In addition, these compounds also show potential in combination therapy. For example, in colorectal cancer, ferroptosis inducers such as **dihydroartemisinin** can be combined with immunotherapy to enhance anti-tumor immune responses by inducing immunogenic ferroptosis [66]. In KRAS mutant cancers, inducers such as **RSL3** can target drug-resistant cells, providing new strategies for treatment [62]. These studies provide an important basis for the application of compounds in cancer treatment and other fields and promote the development of related therapeutic strategies (Table 1).

Ferroptosis inducers such as erastin and RSL3 induce ferroptosis mainly by interfering with the glutathione metabolic pathway. They inhibit the cystine–glutamate reverse transporter (System Xc^−^), reduce the uptake of cystine by cells, and lead to the obstruction of glutathione synthesis. Reduced glutathione levels reduce the activity of glutathione peroxidase 4 (GPX4), which cannot effectively clear lipid peroxides, triggering the accumulation of lipid peroxidation and ultimately activating the intracellular death program related to ferroptosis, resulting in ferroptosis.

## 4. Research on Ferroptosis Inhibitors: Creation, Activity, and Modification

### 4.1. Design and Synthesis of Novel Ferroptosis Inhibitors

With the deepening of the research on ferroptosis, inhibitors based on the structural modification of natural products continue to emerge, bringing new hope for the treatment of diseases [9,42,63,64,74,75,76,77,78,79,80,81,82,83,84,85,86,87,88,89,90,91,92,93]. As a classic inhibitor, ferrostatin-1 and its analogs have been widely studied. Compound **18,** modified by benzenesulfonyl groups, has better inhibitory activity than ferrostatin-1 and shows good solubility and metabolic stability in rat plasma [93]. Gallic tannic acid reacts with 3′-O-amination, which enhances free radical capture, indirect free radical capture, and the inhibitory effect of ferroptosis [84]. Natural products isolated from plants also provide inspiration for the development of inhibitors. Compounds such as **ADC**, isolated from *Ajuga nipponensis*, inhibit ferroptosis by scavenging free radicals and activating the NRF2-AREs pathway [90]. Phloroglucinol compounds found in *Hypericum japonicum* have anti-ferroptosis activity [87]. In addition, the structural modification of other natural products has also achieved results. Compound **7j**, with a phenothiazine-based scaffold design, reduces hERG inhibition while maintaining high ferroptosis inhibitory activity [64]. These inhibitors, based on the structural modification of natural products, with their optimized properties, have shown great potential in the treatment of ferroptosis-related diseases.

In the research and development of ferroptosis inhibitors, compounds designed based on specific chemical structures have shown remarkable results [42,56,64,78,79,82,85,92,93]. Among 2-vinyl-10*H*-phenothiazine derivatives, compound **7j** is prominent. While maintaining the inhibitory activity of high ferroptosis, it can significantly reduce the inhibitory activity of hERG and can also relieve DOX-induced cardiomyopathy. It has good pharmacokinetic properties and provides potential lead compounds for drug discovery targeting ferroptosis [64]. Diarylamine-based organoselenium compounds have antioxidant and anti-ferroptosis activities. This class of compounds has exhibited good anti-iron-promoting activity in 4-tamoxifen-induced GPX4 knockout cell lines, preventing the accumulation of phospholipid hydroperoxides in biofilms [56]. In addition, derivatives such as **olanzapine**, which were designed for structural optimization, such as compound **36**, have significantly improved ferroptosis inhibitory activity compared with olanzapine and are less cytotoxic, making them worthy of development for the treatment of ferroptosis-related neurological diseases [82]. There is also a cannabinol analog, **CP1-CP4**, developed through a fragment-based drug discovery strategy, which retains neuroprotective and mitochondrial regulatory activity and has better efficacy in vivo [42]. These inhibitors, designed based on specific chemical structures, offer new hope for the treatment of ferroptosis-related diseases due to their unique properties.

Nanotechnology-based inhibitors have brought new breakthroughs in the treatment of ferroptosis-related diseases [75,94,95,96,97,98]. UAMC-3203 nanoparticle solution has good stability. In the treatment of corneal injury, it has a high concentration and distribution coefficient in the corneal epithelium, which improves solubility and targeting and shows good therapeutic prospects [75]. In ischemic stroke, GluAC4A carries liproxstatin-1, which enhances solubility. Brain targeting is achieved by glucose modification, and azo groups are released at the ischemic site, reducing ferroptosis and other problems [94]. In the treatment of spinal cord injury, the nanoparticle system combining human umbilical cord mesenchymal stem cells with Feborastatin-1 improves drug utilization, inhibits ferroptosis and inflammatory responses, and promotes neurological recovery [95]. ConA-MelNP nanoparticles targeted at a lesion site can chelate iron ions, which shows good biosafety and therapeutic effects in the treatment of retinal diseases [96]. TAT-MK3b nanoparticles can accumulate in renal tubular epithelial cells, target kidneys, have ROS clearance, reduce ferroptosis, and are beneficial for the treatment of renal injury [98].

### 4.2. Ferroptosis Inhibitory Activity of Known Compounds

**Figure 3:**
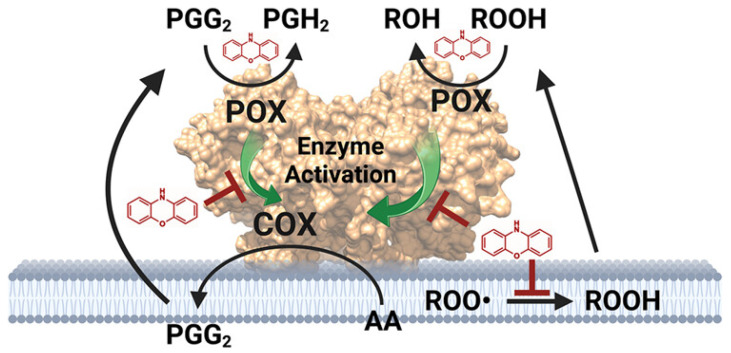
Schematic of ferroptosis inhibitors suppressing prostaglandin synthesis in LPS-stimulated macrophages—reported by Aleem et al. [100]. Reproduced with permission from Ref. [100]. Copyright 2023, American Chemical Society.

The inhibitory effects and mechanisms of ferroptosis in many compounds continue to be studied in depth, providing strong support for the treatment of diseases [9,39,40,42,45,46,50,74,76,88,89,90,94,96,99,100,101,102,103,104,105,106,107,108,109,110,111,112,113,114,115,116,117,118,119] (Figure 3). Olanzapine and its derivatives exhibit the strong inhibitory activity of ferroptosis. The inhibitory effect of its derivative **36** is significantly improved, and cytotoxicity is reduced. The mechanism of action involves antioxidant capacity and is expected to be used in the treatment of ferroptosis-related neurological diseases [82]. As a dual inhibitor of necrosis and ferroptosis cell death pathways, nigretin opens up new avenues for the treatment of complex necrosis-related diseases [40]. Deferramine chelates iron and plays a key role in the treatment of diseases such as pulmonary fibrosis, such as idiopathic pulmonary fibrosis, which slows down. In subarachnoid hemorrhages, it can protect mitochondrial function, improve lipid peroxidation, and reduce neurological deficits and cerebral edema [101]; in spinal cord injury, it can inhibit the ferroptosis of oligodendrocytes [104]; in metabolic dysfunction-related fatty liver disease, it can block ferroptosis markers and improve lipid synthesis/oxidation gene expression [117]. Ferrostatin-1 also plays a key role in the construction of drug-releasing nanoparticle systems in spinal cord injury research [95]. In addition, as in systemic lupus erythematosus, ferroptosis inhibitors may be involved in regulating disease progression, inhibiting ferroptosis, or may be a potential treatment [119]. In noise-induced hearing loss, ferrostatin-1 can inhibit ferroptosis and apoptosis and reduce hearing loss [112]. These studies further clarify the inhibitory effect and mechanism of these compounds, providing more bases and directions for the treatment of related diseases.

### 4.3. Optimization and Modification of Ferroptosis Inhibitors

In the research process for ferroptosis inhibitors, the structural optimization strategy has achieved remarkable results, aiming to improve their performance for better clinical application [64,79,81,85,93,120]. A series of 2-vinyl-10H-phenothiazine derivatives derived from phenothiazine scaffolds has achieved remarkable results. Among them, compound **7j** has been carefully designed to reduce the inhibitory activity of hERG while retaining and enhancing the inhibitory ability of ferroptosis. Its excellent pharmacokinetic properties make it perform well in both in vivo and in vitro experiments. It has become a lead compound for the development of targeted ferroptosis drugs, providing a valuable example for subsequent research [64]. Important breakthroughs have also been made in the modification of iron inhibitor-1 analogs. By modifying its structure, such as that of compound **18**, researchers have shown its stronger potency in inhibiting ferroptosis than the original compound, and its solubility and metabolic stability in rat plasma have also been significantly improved. This achievement makes iron statin-1 analogs more promising in the treatment of ferroptosis-related diseases, making this a solid step toward clinical application [93]. In addition, the value of structural optimization is also reflected in the study of other compounds. For example, in a study of formylpiperazine-derived ferroptosis inhibitors, through the continuous exploration of structures, some compounds were found to have good anti-ferroptosis activity and excellent performance in microsomal stability, providing a new direction for the development of new and efficient ferroptosis inhibitors [81]. Through in-depth study and the optimization of compound structures, not only have the activity, stability, and selectivity of inhibitors been improved but a solid foundation for their wide application in clinical treatment has also been established, which is expected to bring new hope for the treatment of ferroptosis-related diseases (Table 2).

The ferroptosis inhibitors are represented by ferrostatin-1, which mainly plays an inhibitory role by activating the Nrf2 signaling pathway. Ferrostatin-1 can promote the translocation of Nrf2 from the cytoplasm into the nucleus; bind with antioxidant response elements (AREs); and initiate the transcriptional expression of a series of antioxidant genes, such as heme oxygenase-1 (HO-1). These antioxidant proteins enhance the antioxidant defense ability of cells, inhibit lipid peroxidation, and block the occurrence of ferroptosis, thereby protecting cells from the damage of ferroptosis inducers.

## 5. Ferroptosis and Disease Treatment

### 5.1. Ferroptosis Application in Cancer Treatment

For cancer treatment, ferroptosis inducers or inhibitors with other therapies are becoming a research hotspot, opening a new direction for improving their therapeutic effects. In breast cancer treatments, studies have found that the synergistic effect of NR5A2 and NCOA3 can induce breast cancer to develop resistance to BET inhibitors. Ferroptosis can be alleviated by upregulating NRF2, and inhibiting NR5A2/NCOA3 combined with BETi may emerge as a novel approach to breast cancer treatment [131]. The dual PI3K/HDAC inhibitor **BEBT-908** can induce immunogenic ferroptosis in cancer cells and enhance the effect of cancer immunotherapy [132]. Studies on tongue cancer have revealed that the histone deacetylase inhibitor **quicinostat** is highly effective in suppressing the viability, growth, and migration of tongue squamous cell carcinoma cells. Simultaneously, it triggers apoptosis, pyroptosis, and ferroptosis. In animal experiments, it notably curbs the growth of tumor tissues as well, suggesting that it holds promise as a prospective drug for treating tongue squamous cell carcinoma [133].

For lung cancer, immune checkpoint inhibitors have achieved some success in cancer treatment, but some patients are resistant. Studies have found that ferroptosis has a molecular connection to immunotherapy, such as with cisplatin, which can induce ferroptosis in tumor cells, turning “cold” tumors into “hot” tumors, suggesting that the combined application of a ferroptosis activator and epidermal growth factor receptor mutant NSCLC chemotherapy may be an effective strategy in overcoming drug resistance [134]. Ferroptosis has an impact on immune regulation. It alters immune cell function, such as ferroptosis in macrophages, which affects the secretion of inflammatory factors, and ferroptosis in T lymphocytes, which interferes with the immune response. Ferroptosis also affects the immune microenvironment, and substances released by ferroptosis in tumor cells recruit immune cells. In addition, ferroptosis participates in the regulation of inflammatory responses, limiting the spread of inflammation in moderation and exacerbating inflammation in excess.

In hepatocellular carcinoma, multiple studies have shown the potential of combination therapy under different mechanisms. For example, the combination of the deubiquitinase inhibitor **PR-619** with anti-PD1 therapy significantly inhibits colon cancer cell growth, induces ferroptosis, releases damage-related molecular patterns, and triggers anti-tumor immunity [123]. Genetically engineered mouse ferritin co-delivers RSL3 and iFSP1 to tumor tissue, induces immunogenic ferroptosis, and works synergistically with alpha-PD-L1-based immunotherapy to generate a powerful anti-tumor immune response [66]. It has also been found that Sitravatinib, a MerTK inhibitor, sensitizes drug-resistant liver cancer against PD-L1 therapy by promoting tumor ferroptosis and reducing myeloid inhibitory cell infiltration [135].

In addition, studies on other cancers, such as glioblastoma, colorectal cancer, and breast cancer, have also revealed the synergy between different drugs or therapies by modulating the relevant mechanisms of ferroptosis and the combined application of chemotherapy, radiotherapy, and immunotherapy [19,22,30,43,48,51,54,57,59,60,61,62,65,68,70,72,73,136,137,138,139,140,141]. These research results provide more ideas and methods for cancer treatment, which is expected to improve the treatment effect and prognosis of cancer patients.

### 5.2. Application of Ferroptosis in the Treatment of Neurodegenerative Diseases

The emergence of ferroptosis inhibitors has brought new hope for curing neurodegenerative diseases that seriously affect the quality of life of patients and currently have limited treatment options. In a study of Parkinson’s disease, **PTC-041**, as an anti-iron-reducing lipoxygenase inhibitor, could effectively protect fibroblasts derived from patients with primary human Parkinson’s disease from lipid peroxidation and ferroptosis; prevent ferroptosis-related cell nerve loss and astrocyte proliferation in primary rat nerve cell cultures; and also prevent synucleinopathy in vivo [113]. It has also been discovered that apomorphine can suppress ferroptosis, thus pointing to a new avenue for the treatment of Parkinson’s disease [114]. For Alzheimer’s disease, studies have hypothesized that targeting the ferroptosis pathway may be helpful in disease management, such as antifungal cyclopyrrolidine (CPX-O) used alone or in combination with JNK inhibitors in AlCl-induced AD mouse models to improve symptoms—such as the disruption of learning and memory parameters and the degeneration of hippocampal nerve cells—and may be a promising candidate for AD treatment [124].

In the treatment of glaucoma, the p38 mitogen-activated protein kinase (MAPK) pathway inhibitor **SB202190** can inhibit ferroptosis by modulating the ferritin light chain and the SAT1 and SLC7A11/Gpx4 pathways and protect retinal ganglion cells, promising to become a potential retinal protector [53]. In spinal cord injury studies, a variety of ferroptosis inhibitors have shown positive effects. For example, the ferroptosis inhibitor liproxstatin-1 alleviates neurological deficits and brain edema after subarachnoid hemorrhage, reduces nerve cell death, and restores redox balance [101]. Compounds isolated from *Uncaria japonica* have shown inhibitory activity in erastin-induced ferroptosis, in which 3β,6α,23-trihydroxy-olean-12-en-28-oic acids resisted ferroptosis significantly by activating the Nrf2/SLC7A11/GPx4 axis [90]. A synergistic drug-releasing nanoparticle system made of human umbilical cord mesenchymal stem cells and ferroptosis inhibitors was constructed, which significantly inhibited ferroptosis and inflammation after SCI. It promotes the recovery of neurological function [95].

Furthermore, research has revealed that ferroptosis is involved in neonatal hypoxic–ischemic encephalopathy (NHIE), and related compounds play a potential therapeutic role in NHIE by inhibiting ferroptosis [5]. The cannabinol analog **CP1-CP4**, obtained by fragment screening, has an inhibitory effect on oxidation/ferroptosis in nerve cell models, identifying a key molecular scaffold that contributes to neuroprotection [42]. Studies of spinal cord injury have shown that ferroptosis leads to secondary injury, and ferroptosis inhibitors have the potential to treat spinal cord injury [97]. There are also studies screening multiple ferroptosis inhibitors from FDA-approved drugs, providing new possibilities for the treatment of related neurological diseases [9]. These studies demonstrate that ferroptosis inhibitors have great potential in the treatment of neurodegenerative diseases, promising to provide an effective strategy for neuroprotection.

### 5.3. Application of Ferroptosis in the Treatment of Cardiovascular Diseases

Ferroptosis inhibitors have brought new hope for the treatment of cardiovascular diseases. In studies related to myocardial infarction and heart failure, ferroptosis is regarded as being intimately tied to the onset and progression of diseases. Suppressing ferroptosis in the heart is anticipated to serve as an efficacious means for treating cardiovascular ailments. For example, GSK-J4 has the capacity to relieve the hypersensitivity to ferroptosis triggered by palmitic acid by suppressing H3K27 demethylation, thereby reducing oxidative stress and lipid peroxidation, which has potential value in the treatment of hyperlipidemia-induced cardiomyocyte damage [28]. Gliflozine drug empagliflozin can inhibit iron droop under hyperglycemia conditions by restoring the expression of GPX4, enhance skeletal muscle cell survival and paracrine function, and provide a new therapeutic idea for cardiovascular problems caused by diabetic hind limb ischemia [71].

Iron overload cardiomyopathy studies have found that ferroptosis may be the main form of cardiomyopathy, and the ferroptosis inhibitor ferrostatin-1 is more effective than other drugs in reducing mitochondrial dysfunction and improving cardiac function. The inhibition of ferroptosis may be a new treatment for iron overload cardiomyopathy [46]. There are various causes of liver iron overload. Genetic factors such as genetic mutations in hereditary hemochromatosis can cause an imbalance in iron absorption regulation; non-genetic factors include long-term massive blood transfusion and high iron intake. Iron overload causes oxidative damage and inflammatory reactions in the liver, which, in turn, lead to liver fibrosis and even cirrhosis. Diseases such as hemochromatosis and beta-thalassemia often lead to liver iron overload and related complications due to abnormal iron metabolism or blood transfusion.

In terms of aortic dissection, studies have shown that **BRD4770** can act as a novel ferroptosis inhibitor, reducing aortic dilation and reducing morbidity and mortality by inhibiting inflammatory response, lipid peroxidation, and ferroptosis, providing the possibility for the targeted treatment of SMC ferroptosis in aortic dissection [121]. **SP2509** is capable of safeguarding vascular smooth muscle cells against ferroptosis that is induced by diverse stimulations. It achieves this by diminishing intracellular iron concentrations, warding off lipid peroxidation, and averting cell death, and it is expected to relieve aortic dissection [122].

In addition, in research on contrast-induced acute kidney injury (which has a certain association with cardiovascular disease), metallothionein (MT)-related regulation has been found to be related to renal tubular ferroptosis. A combination of zinc acetate and LATS1 inhibitors can upregulate Mt1 expression, which has a protective effect on contrast-induced tubular iron droop, which also provides a reference for the treatment of cardiovascular disease-related kidney injury [142]. Polydopamine nanoparticle-loaded ferrostatin-1 can inhibit ferroptosis in cardiomyocytes by inhibiting the level of NOX4 in cardiomyocytes and reducing the expression of GPX4 and FTH1, thereby inhibiting the ferroptosis of cardiomyocytes and alleviating myocardial ischemia–reperfusion injury [143]. The diamine derivative compound **36**, synthesized based on ferrostatin-1, exhibits good anti-iron apoptotic activity and stability [120].

Overall, ferroptosis inhibitors have shown great potential in the field of cardiovascular disease treatment, but further research on their mechanism of action, optimization of drug performance, and more clinical studies are needed to promote their wide application in the treatment of cardiovascular diseases.

### 5.4. Application of Ferroptosis in the Treatment of Other Diseases

In the research process of various diseases, the mechanism of action of ferroptosis has been continuously examined, opening new directions for disease treatment. In diabetes, ferroptosis is related to beta cell death and insulin resistance, and understanding its mechanism can help to develop new therapeutic strategies. Ferroptosis inhibitors are expected to be potential methods for the treatment of diabetes and its complications [4]. In the death of pancreatic cells in diabetes, ferroptosis is complex. On one hand, oxidative stress, glutathione depletion, and iron overload caused by high glucose can induce ferroptosis and lead to cell damage. On the other hand, cells have their own defense mechanisms, and there are intervention measures in research that can inhibit ferroptosis. In general, although there are inhibitory factors, the diabetic state often makes the balance lean toward ferroptosis induction, resulting in the loss and dysfunction of pancreatic cells.

Pulmonary fibrosis-related studies have shown that deferramine, a ferroptosis inhibitor, can reverse the pulmonary fibrosis phenotype and improve survival in mice. Idiopathic pulmonary fibrosis (IPF) is closely related to ferroptosis. Treating IPF by delivering ferroptosis inhibitors is a promising strategy, such as through the use of pulmonary drug delivery systems to deliver nanomedicines [105]. In ulcerative colitis studies, it has been found that ferroptosis in macrophages plays a role in this disease, and β-caryophyllene inhibits ferroptosis in macrophages and its induced inflammatory response, providing a new target for UC therapy [130]. In acute pancreatitis, the Soat2 inhibitor avasimibe protects mice from AP by inhibiting ferroptosis, suggesting the importance of Soat2 in AP development [111]. For asthma, chicory acid can prevent ferroptosis by inhibiting ALOX15; improve asthma symptoms in mice; and provide new potential drugs and targets for asthma treatment [86]. Acne studies have found that clobetasol propionate can enhance the radiosensitivity of lung cancer cells by inducing ferroptosis, suggesting that it can be used as a radiosensitizer for Keap-1 mutant lung cancer [127].

In investigations of acute kidney injury (AKI), as well as contrast agent-triggered acute kidney injury (CI-AKI), fullerenol nanoparticles can prevent ferroptosis in cisplatin-induced AKI by inhibiting renal lipid peroxidation and ferrous accumulation. The combination of zinc acetate and LATS1 inhibitors can improve CI-AKI, indicating a key role for ferroptosis in the pathogenesis of AKI and providing new targets for treatment [128,142]. Dry eye studies have shown that the DDR1 inhibitor **DDR1-IN-1** can improve dry eye symptoms by inhibiting iron deposition, emphasizing the importance of DDR1 in dry eye [144]. In low back pain studies, tenoredine inhibits ferroptosis in nucleus pulposus cells and alleviates the progression of intervertebral disc degeneration in rats, providing support for the treatment of low back pain from the perspective of ferroptosis inhibition [91].

In addition, in studies of other diseases, such as polycystic ovary syndrome, systemic lupus erythematosus, spinal cord injury, etc., ferroptosis-related mechanisms have also been revealed, such as with iron statin-1, which can reduce homocysteine-induced ovarian granulosa cell damage [50]. Ferroptosis inhibitors may be involved in regulating the development of systemic lupus erythematosus [119]. Ferroptosis inhibitors can improve secondary injury after spinal cord injury [97]. Studies show that the role of ferroptosis in a variety of diseases is gradually becoming clear, providing a theoretical basis for the development of targeted treatment strategies. However, there is still a need to further study its specific molecular mechanisms, optimize treatment plans, and strengthen drug research and development to better translate these findings into clinical practice, bringing more treatment options and hope to patients. At the same time, it is also necessary to pay attention to the specificity and commonality of ferroptosis mechanisms in different diseases to more precisely regulate this process and achieve the effective treatment of diseases (Table 3).

## 6. Conclusions and Outlook

In recent years, ferroptosis, a new type of programmed cell death, has been extensively studied. Research on its mechanisms has reached molecular, pathway, and signaling levels. For example, the NUPR1 inhibitor ZZW 115 triggers ferroptosis through mitochondrial changes, and RSL3’s interaction with TXNRD1 shows the role of redox balance. The HIF-α pathway also affects ferroptosis by regulating lipid-related genes in glioblastoma. In drug development, novel inducers like compound **26a** (targeting GPX4) show strong anti-tumor activity, while inhibitors such as 2-vinyl-10H-phenothiazine derivatives reduce toxicity without sacrificing efficacy. Ferroptosis-based combination therapies have great potential in cancer treatment. Inhibitors also have applications in neurodegenerative and cardiovascular diseases, as seen with GSK-J4 in cardiomyocytes. However, ferroptosis research still has hurdles. Its regulatory network is complex, and drug-related issues like specificity, toxicity, and combination therapy optimization remain. Looking ahead, technologies like single-cell sequencing may clarify the regulatory network, enabling personalized treatments. Future drugs are expected to be more effective and stable, and combination therapies may expand, bringing more treatment breakthroughs.

## Figures and Tables

**Table 1:** Comparison of mechanisms and characteristics of various ferroptosis inducers.

Inducer Compound Name/Type	Mechanism of Action	Related Disease Research	Refs.
RSL3 and ML162	Affect redox balance and promote lipid peroxidation and ferroptosis	Proliferation of leukemia cells	[18,55]
Compound 26a and other targeted GPX4 inhibitors	Targeted inhibition of GPX4	Breast cancer research	[27]
Erastin	Interrupts cystine uptake and depletes intracellular glutathione	Study of leukemia cells	[18]
Synergistic inducers such as dihydroartemisinin and JKE1674	Synergistic effect with ferroptosis inducer to trigger ferroptosis	Breast cancer research	[19]
Sorafenib and other signaling pathway-related inducers	The expression of related transcription factors changes, promoting ferroptosis	Hepatocellular carcinoma and cardiotoxicity studies	[72]
Drug-resistant tumor inducers such as lapatinib	Induced ferroptosis	KRASG12C mutant cancer cells	[62]
MTX-LDH@MnO and other nano platform inducers	Disrupt the metabolic activity of antioxidants and promote ferroptosis	Cancer immunotherapy	[73]
DOX and other chemotherapy drug inducers	Induced by affecting oxidative stress-related pathways	Cardiomyopathy research	[64]
1-(4-(4-Methylpiperazin-1-yl) phenyl) ethyl-10H-thiazide (51) and other new inducers	The mechanism of action is not mentioned in detail	Ischemic stroke model studies	[29]
9D and other novel hybrid triazine inducers	Induce lipid peroxidation and initiate ferroptosis	Colorectal cancer research	[61]

**Table 2:** Summary and comparison of ferroptosis inhibitors.

Type of Inhibitor	Representative Compounds/Inhibitors	Overview of Mechanism of Action	Refs.
Free radical capture antioxidants	Ferrostatin-1 and its analogs	Capture free radicals	[31,81,93,104,112]
Mitochondrial protectant	Liproxstatin-1	Protects mitochondrial function;	[101,104,117]
Iron-chelating agent	Deferriamine	chelates iron ions	[46,105]
Active oxygen scavenger	N-Acetylcysteine	Removes reactive oxygen species	[110,115]
Enzyme inhibitor	PD146176	Inhibits specific enzyme activity	[86]
Analogs/metabolites/combination effects/synergistic enhancement/synergistic weakening inhibitors	Idebenone, oleic acid, kinofen, SCD1 inhibitor, zinc protoporphyrin-9	Regulate metabolic influence pathway	[17]
Antioxidant capacity inhibitors	Olanzapine and its derivatives	Antioxidant capacity	[82]
Multipurpose inhibitor	UAMC-3203	Effects on corneal injury, liver injury, etc.	[75,99]
Multifunctional inhibitor	Necrostatin-1	Inhibits a variety of cell death-related proteins	[100]
Histone methyltransferase inhibitors	BRD4770	Regulates histone methylation	[121]
Lysine-specific demethylase 1 inhibitors	SP2509	Reduces intracellular iron levels	[122]
Deubiquitin enzyme inhibitors	PR-619	Degrades related proteins	[123]
Inflammatory body inhibitors	MCC950	Inhibits the inflammatory body	[118]
Nrf2-related inhibitors	Tinoridine	Binds to Nrf2 and promotes its expression and activity	[91]
Multifunctional regulator	Melatonin	Anti-inflammatory, iron chelator, and antioxidant	[10]
Iron-chelating neuroprotectant	CPX-O	Iron chelation and regulation of related pathways	[124]
Autophagy pathway-targeting inhibitors	KW-2449	Targets the autophagy pathway	[41]
NOX4 inhibitors	GLX351322	Inhibits NOX4 activity and inhibits iron sagging	[125]
Multifunctional inhibitor	Formylpiperazine-derived compounds	Multifaceted regulation of cell state	[81]
Antioxidant activator	Dithiothiones	Activate transcription factors; upregulates GSH levels	[3]
Enzyme inhibitor	TPPU	Inhibits specific enzymes	[126]
Multimechanism inhibitor	Hinokitiol	Chelates iron and activates transcription factors; upregulates antioxidant genes	[45]
Covalent-binding inhibitor	GPX4 inhibitors	Covalently bind to GPX4-specific locations	[12]
Structure optimization inhibitors	NecroX-7 and eriodictyol-7-*O*-glucoside	Modify structure-related parameters	[85]
Lipoxygenase inhibitors	PTC-041	Inhibits specific lipoxygenase	[113]
Dopamine-receptor-independent inhibitors	Apomorphine	Inhibits lipid peroxidation	[114]
Inhibitors of signaling pathway regulation	Valproic acid	Inhibits specific signaling pathways and induces ferroptosis	[127]
Free radical-scavenging inhibitors	Fullerenol nanoparticles	Use free radical scavenging	[128]
Gene regulatory inhibitors	miR-3587 inhibitors	Regulate gene expression and inhibit ferroptosis	[129]
4-Hydroxypyrazole derivatives (e.g., HW-3, Compound **25**)	Antioxidant through free radical capture; inhibits ferroptosis	Ferroptosis inhibition and related diseases	[63]
Disease-related inhibitors	Soat2 inhibitors	Inhibit ferroptosis and improve disease Symptoms	[111]
Receptor activation inhibitor	Beta-Caryophyllene	Activates receptors	[130]

**Table 3:** The role of ferroptosis in disease treatment.

Disease Type	The Role of Ferroptosis in Disease	Specific Diseases	Refs.
Cancer	Inhibits tumor growth and has great potential for combined application with chemotherapy, radiotherapy, and immunotherapy	Breast cancer, lung cancer, liver cancer, and other cancers	[19,22,30,43,48,51,60,62,66,70,72,113,123,131,132,133,135,136,137,138,139,141,145]
Neurodegenerative diseases	Associated with nerve cell death and neurological impairment; inhibition of ferroptosis provides neuroprotection	Parkinson’s disease, glaucoma, spinal cord injury, and neonatal hypoxic-ischemic encephalopathy	[5,7,9,23,42,45,53,90,95,97,101,104,109,112,113,114]
Cardiovascular disease	Inhibiting ferroptosis is of great significance for the treatment of cardiovascular diseases	Myocardial infarction, heart failure, aortic dissection, and contrast-induced acute kidney injury	[28,46,71,120,121,122,142,143]
Other diseases	Intervention against ferroptosis brings new targets and ideas for the treatment of these diseases	Diabetes, pulmonary fibrosis, ulcerative colitis, acute pancreatitis, asthma, dry eye, low back pain, acne, acute kidney injury, and contrast-induced acute kidney injury	[4,10,11,50,52,85,91,92,105,110,111,115,117,124,125,126,127,128,130,144,146,147,148,149,150,151]
